# Bi-directional associations between gender-based harassment at work, psychological treatment and depressive symptoms

**DOI:** 10.3389/fpsyg.2023.1278570

**Published:** 2023-11-29

**Authors:** Johan Paulin, Paraskevi Peristera, Anna Nyberg

**Affiliations:** ^1^Department of Public Health and Caring Sciences, Uppsala University, Uppsala, Sweden; ^2^Stress Research Institute, Department of Psychology, Stockholm University, Stockholm, Sweden

**Keywords:** gender-based harassment, discrimination, sexism, depressive symptoms, psychological treatment, structural equation models

## Abstract

**Introduction:**

The objective of this study was to investigate the bi-directional associations between experienced and witnessed gender-based harassment (GBH) on the one hand, and depressive symptoms and psychological treatment on the other, in an occupational setting. GBH are behaviors that derogate, demean, or humiliate an individual based on his or her gender.

**Methods:**

The analyses were based on data from the Swedish Longitudinal Occupational Survey of Health at 2018 (T1) and 2020 (T2), including 6,679 working participants (60.3% women) with a majority in the age range of 45–64. Using cross-lagged structural equational models, we analyzed experienced and witnessed GBH in relation to depressive symptoms and having received psychological treatment (talked to a counselor or psychological professional) over time.

**Results:**

Our results showed that neither experienced nor witnessed GBH was prospectively associated with depressive symptoms or psychological treatment over two years. Both higher levels of depressive symptoms (β = 0.002, *p* ≤ 0.001) and having received psychological treatment (β = 0.013, *p* = 0.027) weakly predicted experiences of GBH over time. Having received psychological treatment was furthermore weakly associated with witnessed GBH (β = 0.019, *p* = 0.012).

**Discussion:**

In conclusion, the hypothesized associations between exposure to GBH and mental health outcomes were not statistically significant, while a weak reverse association was noted. More research addressing bidirectional associations between GBH and mental health outcomes are needed.

## Introduction

Gender-based harassment (GBH) in the workplace, either alone or together with other demeaning behaviors, is one of the most frequent yet elusive work environment problems to address (Charney and Russell, 1994; Mazzeo et al., 2001; Langhout et al., 2005; Leskinen et al., 2011; Quick and McFadyen, 2017). GBH is a broad concept and includes harassment expressed as jokes, attitudes about stereotypical gender roles, dismissive or undermining jargon relating to competence or experience, and more general behaviors such as insulting, degrading, or contemptuous attitudes related to sex and gender identity (Fitzgerald et al., 1988, 1995; Leskinen et al., 2011). In the Swedish work environment survey, gender-based harassment was reported by 7.5% of the working population in 2007–2013 and has been found to be associated with register-based sickness absence (Blindow et al., 2021) and use of psychotropic medication (Blindow et al., 2022) in this population. In the present study, we have chosen to use the term gender-based harassment, which builds on Berdahl's (2007) term *sex-based harassment*, which is defined as behaviors that derogate, demeans, or humiliate an individual based on that individual's sex (Berdahl, 2007). Berdahl argues that the main motivator for harassment is the individuals' desire to protect and uphold their own sex-based status. GBH does the same but is based on an individual's gender, not the only two sexes legally recognized in Sweden. The motivation behind GBH concerns not only the harassers' personal gain but also their assimilation of societal, sexist gender hierarchies that dictate what is acceptable or not. All sexes and genders can experience punishment and harassment if not conforming whether they are demure or powerful, physically strong or weak, in majority or minority at the workplace. The context and societal norms dictate how individuals threatening to gender stereotypes are perceived and to what extent they risk being subjected to harassment. Women working in male-dominated fields and men working in female-dominated fields have been at increased risk for harassment (Willness et al., 2007; Rospenda et al., 2009; McLaughlin et al., 2012; Quick and McFadyen, 2017). Other risk groups are women in high-ranking positions (Folke et al., 2020) and those who do not fall within traditional gender stereotypes, i.e., women being more outspoken, active and assertive, feminist, or sexually non-conforming (Konik and Cortina, 2008; Holland and Cortina, 2013; Leskinen et al., 2015; Cortina and Areguin, 2021). Men are not exempt from the risk of being harassed, more frequently being targets of harassment if engaging in feminist activism or not conforming to heteronormative stereotypes (Konik and Cortina, 2008; Berdahl and Moon, 2013; Holland et al., 2016).

Much of the research on GBH has focused on direct exposure primarily to gender harassment, but another aspect that is also potentially harmful is to witness others being targets of GBH, as expressed with or without sexual content (Glomb et al., 1997; Dionisi and Barling, 2018; Takeuchi et al., 2018; Benzil et al., 2021). Despite the individual not being the primary target of the harassment, witnessing GBH may elicit a range of emotional responses, ranging from an empathetic response in identifying with the person experiencing the harassment to anger, fear, or worry for personal safety in the work environment (Powell, 2011; McMahon and Banyard, 2012; Gabriel et al., 2023).

Most of the research conducted within the field of GBH exposure at work has used sexual harassment as the main construct, with some including GBH within that construct. Several cross-sectional studies have documented associations between sexual harassment and depressive symptoms (Marsh et al., 2009; Hanson et al., 2015; Friborg et al., 2017; Adler et al., 2021) and a minority of the broader concept of GBH (van Roosmalen and McDaniel, 1999; Vargas et al., 2020). GBH has also been linked to other mental health outcomes such as burnout (Takeuchi et al., 2018), post-traumatic stress disorder, and anxiety (Willness et al., 2007). When it comes to longitudinal studies, only a few investigated the mental health implications of GBH. Houle et al. (2011) found that being exposed to sexual harassment in an early career stage (ages 19–26) predicted increased depressive symptoms at age 30–31 years. A Norwegian study (Sterud and Hanvold, 2021) found that sexual harassment was a significant predictor of mental distress over a time of 3 years. Reverse associations were investigated but not found to be statistically significant. In a large Danish longitudinal study (Rugulies et al., 2020) following participants from 2012 to 2016, the authors found an association between sexual harassment in 2012 and elevated depressive symptoms 2 years later. The association had increased in strength at a third measurement 4 years after baseline. Another recent longitudinal study from South Korea reported a statistically significant association between sexual harassment by clients and self-reported symptoms of burnout (Jung and Yoon, 2020). Glomb et al. (1999) and Munson et al. (2000) investigated the broader concept of GBH on 216 women working in academia and found that GBH affected psychological wellbeing and distress over a 2-year time lag.

The association between GBH and psychological treatment is not clearly established. A longitudinal study found an association between sexual harassment and the use of any type of services, including physical, psychological, or spiritual (Rospenda et al., 2006). In a second study by the same authors (Shannon et al., 2007), it was found that the effect of service use varied when broken down into different types of services. They found no significant association between sexual harassment and seeking mental health services; however, significant results were found for seeking legal and spiritual services. In contrast to the above studies, the present study investigates the wider concept of gender-based harassment, i.e., not only sexual harassment, in relation to specifically seeking psychological treatment, both short- and long-term.

Even though witnessing GBH at work is fairly common, only a few studies have investigated the health effects of such exposure (Glomb et al., 1997; Ford et al., 2021), with the majority focusing on sexual harassment (Schneider et al., 1997; Benzil et al., 2021). In cross-sectional studies, witnessing sexual harassment has been associated with mental ill-health, such as burnout, anxiety, and depression (Richman-Hirsch and Glomb, 2002; Miner-Rubino and Cortina, 2007; Miner and Eischeid, 2012; Takeuchi et al., 2018). One study reported that men and women witnessing GBH toward women were more likely to find the organization unresponsive toward dealing with such behavior, which in turn was associated with lower wellbeing in these men and women (Miner-Rubino and Cortina, 2007). A study by Dionisi and Barling (2018) found that the link between witnessing male gender harassment and mental health symptoms was mediated by anger. As far as we know, there is a lack of studies examining the mental health effects of witnessing GBH over time.

Summarizing previous research, we have identified several areas where additional research is needed. First of all, most studies focus on sexual harassment. Research on the health consequences of harassment that the affected perceive as based on their gender is still largely lacking. Furthermore, individual exposure to GBH is often investigated as a separate concept, with few studies focusing on witnessing others being exposed to GBH (Bowes-Sperry and O'Leary-Kelly, 2005; McMahon and Banyard, 2012; McDonald et al., 2016; Quick and McFadyen, 2017). Additionally, reverse causation has not been investigated in most previous longitudinal research so far. A systematic review (Tang, 2014) of 10 studies found evidence suggesting that associations between psychosocial work environment exposures and mental health outcomes often follow a reciprocal process in which employee mental health appears to play a role in subsequent reports about their work environment. While it is unclear if these findings are driven by worse mental health leading to stronger negative perceptions of experiences or if employees with poor mental health are more prone to be targets of abuse, the reverse associations between GBH and mental ill-health require more attention in research. Other limitations are that only self-reported symptoms of poor mental health have been used as health indicators in most previous studies, whereas we will include reports of treatment as an outcome variable. In a recent study, we found an association between self-reported sexual and gender harassment and the prospective use of psychotropic medication (Blindow et al., 2022). However, in Sweden, when seeking healthcare for mild-to-moderate depression or anxiety disorders, psychological treatment is the first treatment option that should be offered to the patient ahead of psychotropic medication, according to National Guidelines for Depression and Anxiety (National Board of Health and Wellfare, 2021). Reports of having sought psychological treatment are there for a realistic first indication of mental health problems that are so severe and persistent that the affected sought professional help.

In the present study, we will investigate the longitudinal associations between experienced and witnessed GBH as exposure variables, and self-rated depressive symptoms and psychological treatment as outcome variables. The overall aim is to investigate the following specific research questions: Is there an association over time between (a) experienced gender-based harassment and depressive symptoms? (b) experienced gender-based harassment and receiving psychological treatment? (c) witnessed gender-based harassment and depressive symptoms? and (d) witnessed gender-based harassment and receiving psychological treatment?

## Materials and methods

### Study design and study sample

A cross-lagged panel design investigated bi-directional associations between the exposures and hypothesized outcomes using two waves (2018 and 2020) of the Swedish Longitudinal Occupational Survey of Health (SLOSH). SLOSH is a longitudinal project that builds on participants of the Swedish Work Environment Surveys (SWES, 2003–2011). SWES is a nationally representative sample of the Swedish working population whose participants are invited to participate in the SLOSH cohort. The survey has been conducted biennially since 2006 and consists of a self-completion questionnaire in two versions, one for those who work 30% or more full-time and one for those who work less or not at all. SLOSH focuses on connecting various work-related variables with issues about individuals' health and general wellbeing. A detailed description of the SLOSH cohort can be found in Magnusson Hanson et al. (2018), and more additional information can also be found at www.slosh.se.

The overall response rates for the SLOSH waves 2018 (T1) and 2020 (T2) were 48% and 49%, respectively. For our study, we selected participants who worked at least 30% in 2018 and 2020 (*n* = 7546). Due to most self-employed businesses (96%) having <10 employees (The Swedish Agency for Economic and Regional Growth, 2023) and thus at a lower risk of encountering GBH, self-employed respondents (*n* = 867) were excluded, reducing the sample to 6679 individuals (60.29% women and 39.71% men). To be able to separate the effect of witnessing GBH from the effect of experiencing GBH personally, we excluded individuals who reported that they had experienced GBH (*n* = 381) in the analyses of witnessed GBH. This resulted in a sample of *n* = 6298 (58.91% women and 41.09% men). Information on socio-demographic characteristics was obtained from the registered Longitudinal Integration Database for Health Insurance and Labor Market Studies (LISA) and linked to data from the SLOSH questionnaire. The study was approved by the Regional Research Ethics Board in Stockholm (No: 2019-05590).

### Variables

#### Exposure variables

The questions used in the SLOSH study regarding experienced and witnessed gender-based harassment were constructed by the authors in collaboration with an expert on gender harassment. Experienced GBH was measured with three questions about harassment; “Have you during the past 6 months been subject to harassment because of your gender by (a) managers, (b) co-workers, or (c) others (e.g., customers, clients, patients, or students)?”. Witnessed GBH was measured with a single question; “Have you during the past 6 months, heard or seen someone else in the workplace being harassed because of their gender?”. The four response alternatives for all variables ranged from “At least once during the week” to “No”. Due to restrictions in statistical power, the three questions about experienced GBH were combined into one variable, and participants who reported any exposure were categorized as exposed, regardless of the reported frequency and who they identified as the harasser. The variable for witnessed GBH was also dichotomized in the same way.

#### Outcome variables

Depressive symptoms were measured with The Symptom Checklist-core depression (SCL-CD6) (Magnusson Hanson et al., 2014), a brief subscale from the (Hopkins) Symptom Checklist (SCL-90). The respondents were asked “How much during the last week have you been troubled by (a) feeling lethargy or low in energy, (b) feeling blue, (c) blaming yourself, (d) worrying too much, (e) feeling no interest in things, and (f) feeling that everything is an effort”, with five response alternatives ranging from “Not at all” to “Very much”. The depression index score ranged from 0 (lowest) to 24 (highest). Cronbach's alpha for the 2018 wave was α = 0.91 and α = 0.90 for the 2020 wave. The score was used as a continuous measure in our analysis.

Psychological treatment was measured with two questions in the SLOSH questionnaire: “During the last 2 years have you received (a) shorter psychological treatment such as motivational or support interventions with a counselor, and (b) psychotherapy, i.e., longer psychotherapy with, e.g., a psychologist or psychotherapist”. The response options were “Yes” and “No”. Answering “Yes” to either of the two questions were considered as having received psychological treatment.

#### Covariates

The following covariates were retrieved from the SLOSH questionnaires in 2018 and 2020. Workplace violence measured exposure to violence, the threat of violence, and bullying being subjected to personal persecution through mean words and actions during the past 6 months. Both workplace violence and bullying had response alternatives ranging from 1 (Yes, many times during the week) to 4 (No). For our analysis, the alternatives were dichotomized into “No” or “Yes”, where yes indicated being exposed at least once. Job demands and decision authority were measured using the *Swedish Demand Control Questionnaire* (Sanne et al., 2005). Job demands were measured with four items: “Do you have to work very fast?”, “Does your work demand too much effort?”, “Does your work often involve conflicting demands?”, and “Do you have enough time to do everything?” The first three items were reversed before combining. Control at work was measured using two items (“Deciding what to do at work” and “Deciding how to do your work”). Rating scales for all six items ranged from 1 “Yes, often” to 4 “No, hardly ever/never”. Higher values indicate higher job demands and lower decision authority. For this study, higher job demands and lower decision authority were coded as binary variables, with 1 indicating above-the-median job demands and median-or-less job control and 0 otherwise.

The following covariates were retrieved from LISA and linked to SLOSH survey items through the Swedish personal number. Information on participants' gender was available with the options “woman” and “man”. Age was categorized into five groups (<34 years, 35–44, 45–54, 55–64, and >64 years old). Marital status was coded as a dichotomous variable, with 1 indicating married/cohabiting and 0 otherwise. Education was coded as “up to 9 years”, “up to 12 years”, “university education <3 years”, and “university education ≥3 years”. Country of birth was dichotomized as “Swedish” and “Other”. Origin of parents was categorized as “One or both parents born in Sweden” and “Parents born outside Sweden”. Disposable income was categorized into quartiles. Type of occupation was categorized from the Swedish Standard Classification of Occupations (SSYK), which in turn is based on the international standard ISCO-08, and encompasses all occupations on the Swedish labor market and includes nine categories: “Legislators, senior officials, managers”, “Professionals”, “Technicians”, “Clerks”, “Service workers”, “Agricultural and fishery workers”, “Craft workers”, “Machine Operators”, and finally “Elementary occupations”.

### Analytical strategy

#### Cross-lagged SEM models

We employed structural equation modeling (SEM) techniques to fit our data. All analyses were run in STATA using the Stata command SEM. The utilized two-wave autoregressive cross-lagged panel design allows for the examination of the mutual effects of two variables on one another over time with a 2-year time lag. This approach presents several advantages such as the construction of latent variables, simultaneous estimation with correlated residuals, and management of missing data (Little et al., 2007).

We fit four structural equation models to examine the relationship between two types of GBH (Experienced and Witnessed) and the outcomes of depressive symptoms and psychological treatment. Model 1 was an unadjusted, reciprocal model including all autoregressive and cross-lagged paths between the two time points 2018 (T1) and 2020 (T2). This model examined the cross-lagged paths from experienced GBH at T1 to depressive symptoms at T2, the reversed paths from depressive symptoms at T1 to experienced GBH at T2, and the paths from experienced GBH and depressive symptoms at T1 to the same variables at T2. Model 2 extended Model 1 by adjusting for the socio-demographic covariates at T1 (age, gender, education, income, country of birth, type of occupation, civil status, and origin of parents). Model 3 included the workplace covariates, job demands, and job control in addition to the demographic covariates. Model 4 additionally controlled workplace violence and bullying. These four models were fitted separately for experienced and witnessed GBH with the respective outcome variables of depressive symptoms and psychiatric treatment. The fit of the models was assessed via several fit statistics: the root mean square error of approximation (RMSEA), the comparative fit index (CFI), the standardized root mean square residual (SRMR), and Tucker–Lewis index (TLI), with cutoff values recommended for a good fit being RMSEA ≤ 0.08, CFI ≥ 0.90, SRMR ≤ 0.08 and TLI >0.90. Models 1–3 are presented in Appendix. Missing data were handled by full information maximum likelihood (FIML). Benchmark values for effect sizes when interpreting the size of cross-lagged effects of CLPM according to Orth et al. (2022) is 0.03 (small effect), 0.07 (medium effect), and 0.12 (large effect).

## Results

The descriptive statistics for the study variables in SLOSH T1 (2018) and T2 (2020) are presented in Table 1. Of the 179 cases of experienced GBH in T2, 74 had already reported harassment at T1. For witnessed GBH, there were 394 cases at T2, of which 133 were already cases at T1. In both waves, approximately 13% of study participants received psychological treatment over the past 2 years. Of the 899 participants who reported having received psychological treatment at T2, 425 were already in treatment at T1. Depressive symptoms were significantly higher at T1 compared to T2. Statistically significant differences between waves were also found for both exposure variables and the workplace covariates bullying, violence and threats of violence, and job demands.

**Table 1:** Distribution of study variables for the total sample in SLOSH waves 2018 and 2020.

	**2018**	**2020**
	***N*** = **6,679**	***N*** = **6,679**
**Gender (** ***n*** **, %)**		
Women	4,027 (60.29)	4,027 (60.29)
Men	2,652 (39.71)	2,652 (39.71)
**Age (** ***n*** **, %)**		
≤ 34 years	274 (4.10)	149 (2.23)
35–44 years	1,098 (16.44)	918 (13.74)
45–54 years	2,437 (36.29)	2,180 (32.64)
55–64 years	2,690 (40.28)	2,945 (44.09)
>64 years	180 (2.70)	487 (7.29)
**Education (** ***n*** **, %)**		
≤ 9 years	248 (3.72)	244 (3.65)
≤ 12 years	885 (22.59)	2,442 (36.56)
University < 3 years	526 (7.88)	529 (7.92)
University ≥3 years	3,438 (51.51)	3,464 (51.86)
**Marital status (** ***n*** **, %)**		
Not married/cohabiting	2,743 (41.54)	2,783 (41.67)
Married/cohabiting	3,860 (58.46)	3,896 (58.33)
**Income (SEK)**		
≤ 329,999	1,603 (24.00)	1,472 (22.04)
330,000–399,999	1,591 (23.82)	1,732 (25.93)
400,000–509,999	1,781 (26.67)	1,803 (27.00)
≥510,000	1,704 (25.51)	1,672 (25.03)
**Type of occupation (** ***n*** **, %)**		
Legislators, senior officials, managers	672 (10.27)	685 (10.41)
Professionals	2,492 (38.08)	2,515 (38.23)
Technicians	1,158 (17.70)	1,162 (17.66)
Clerks	474 (7.24)	491 (7.46)
Service workers	942 (14.39)	935 (14.21)
Agricultural and fishery workers	27 (0.41)	33 (0.50)
Craft workers	353 (5.39)	355 (5.40)
Machine operators	308 (4.71)	299 (4.55)
Elementary occupations	118 (1.80	103 (1.57)
**Country of birth (** ***n*** **, %)**		
Sweden	6,249 (93.56)	6,249 (93.59)
Other	430 (6.44)	428 (6.41)
**Origin of parents (** ***n*** **, %)**		
One or both parents born in Sweden	6,106 (91.42)	6,106 (91.42)
Parents born outside Sweden	573 (8.58)	573 (8.58)
Depressive symptoms (M, SD)^*^	5.07 (4.99)	5.19 (4.87)
**Psychological treatment (** ***n*** **, %)** ^ns^		
Yes	898 (13.45)	899 (13.46)
No	5,781 (86.55)	5,780 (86.54)
**Experienced gender-based harassment (** ***n*** **, %)** ^***^		
Yes	276 (4.13)	179 (2.68)
No	6,403 (95.87)	6,500 (97.32)
**Witnessed gender-based harassment (** ***n*** **, %)** ^***^		
Yes	535 (8.1)	394 (5.90)
No	6,144 (91.99)	6,285 (94.10)
Job demands (M, SD)^***^	2.58 (0.55)	2.49 (0.55)
Decision authority (M, SD)^ns^	1.92 (0.71)	1.89 (0.72)
**Bullying (** ***n*** **, %)** ^***^		
Yes	530 (8)	1,122 (16.88)
No	6,094 (92)	5,523 (83.12)
**Violence at workplace (** ***n*** **, %)** ^***^		
Yes	640 (9.66)	822 (12.36)
No	5,985 (90.34)	5,826 (87.64)

* *p* < 0.05.

*** *p* < 0.001.

ns, non-significant.

Paired two-tailed t-test of the relationship between respondents in 2018 and 2020.

### Experienced GBH and depressive symptoms

**Figure 1:**
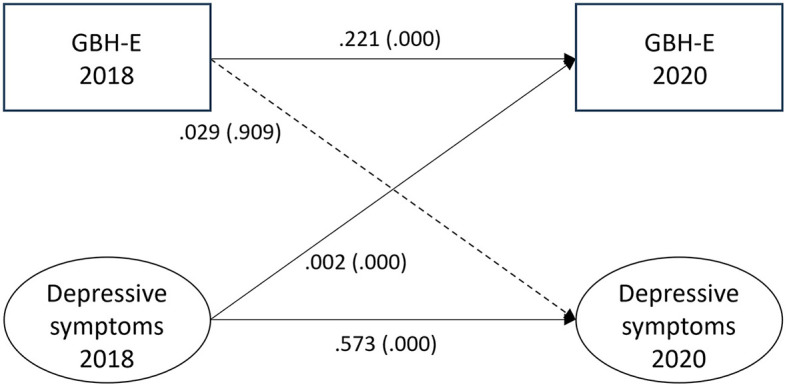
Paths between experienced gender-based harassment (GBH-E) and depressive symptoms (*n* = 6,679) in SLOSH 2018 and 2020. Analyses were adjusted for age, gender, education, income, country of birth, origin of parents, type of occupation, marital status, job demands, job control, violence, and bullying. Statistically significant paths are shown in solid lines.

The estimated paths between experienced GBH and depressive symptoms for the fully adjusted model 4 (adjusted for age, gender, education, income, country of birth, type of occupation, civil status, origin of parents, job demands, job control, workplace violence, and bullying) are shown in Figure 1. There was no association between experienced GBH at T1 and depressive symptoms at T2 (0.029, *p* = 0.909). The estimate for the small reverse association, from depressive symptoms at T1 to experienced GBH at T2, was (0.002, *p* = 0.000). The auto-regressive paths between T1 and T2 for both experienced GBH (0.221, *p* = 0.000) and depressive symptoms (0.573, *p* = 0.000) indicate large effects and within-variable stability across time. The fit statistics were RMSEA = 0.000, CFI = 1.000, SRMR = 0.000, TLI = 0.995.

### Experienced GBH and psychological treatment

**Figure 2:**
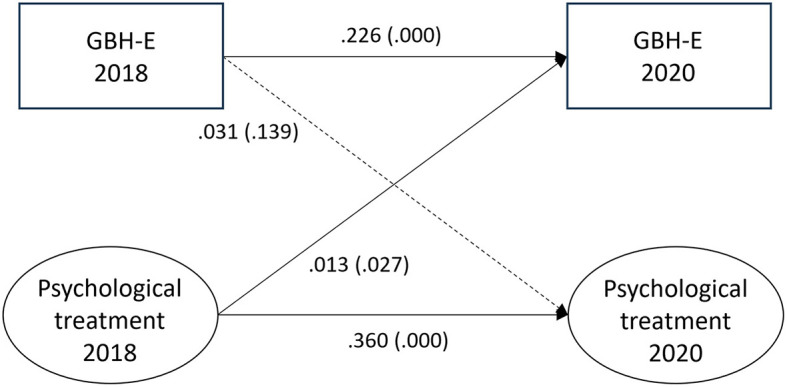
Paths between experienced gender-based harassment (GBH-E) and psychological treatment (*n* = 6,679) in SLOSH 2018 and 2020. Analyses were adjusted for age, gender, education, income, country of birth, origin of parents, type of occupation, marital status, job demands, job control, violence, and bullying. Statistically significant paths are shown in solid lines.

The paths between experienced GBH and psychological treatment are illustrated in Figure 2. For the hypothesized path between experienced GBH at T1 and psychological treatment at T2, the estimate was 0.031 (*p* = 0.139). For the reversed association between psychological treatment and experienced GBH, the estimate was small (0.013, *p* = 0.027). The auto-regressive paths for experienced GBH (0.226, *p* = 0.000) and psychological treatment (0.360, *p* = 0.000) indicate stability in the variables across time. Fit statistics for experienced GBH and psychological treatment were RMSEA = 0.000, CFI = 1.000, SRMR = 0.000, and TLI = 1.000.

### Witnessed GBH and depressive symptoms

**Figure 3:**
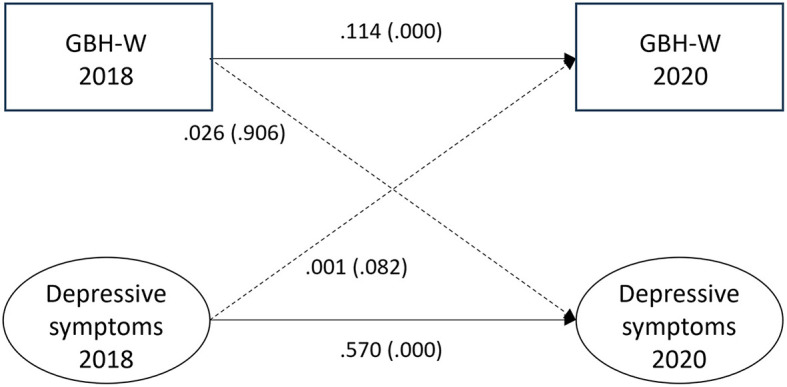
Paths between witnessed gender-based harassment (GBH-W) and depressive symptoms (*n* = 6,298) in SLOSH 2018 and 2020. Analyses were adjusted for age, gender, education, income, country of birth, origin of parents, type of occupation, marital status, job demands, job control, violence, and bullying. Statistically significant paths are shown in solid lines.

The associations between witnessed GBH and depressive symptoms for the fully adjusted model are shown in Figure 3. No association was found for the hypothesized path between witnessed GBH at T1 and depressive symptoms at T2 (0.026, *p* = 0.906) or for the reversed path between depressive symptoms at T1 and witnessed GBH at T2 (0.001, *p* = 0.082). Auto-regressive paths from T1 to T2 were for depressive symptoms 0.570 (*p* = 0.000) and for witnessed GBH 0.114 (*p* = 0.000). Fit statistics for the model were RMSEA = 0.000, CFI = 1.000, SRMR = 0.000, and TLI = 1.000.

### Witnessed GBH and psychological treatment

**Figure 4:**
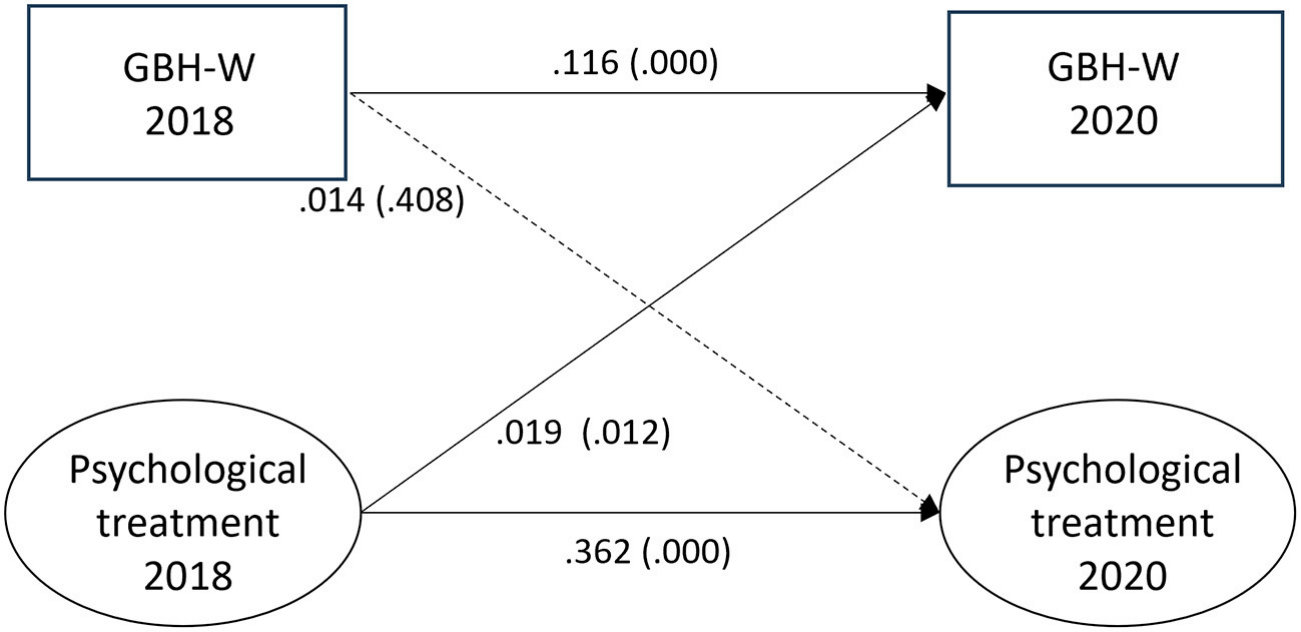
Paths between witnessed gender-based harassment (GBH-W) and psychological treatment (*n* = 6,298) in SLOSH 2018 and 2020. Analyses were adjusted for age, gender, education, income, country of birth, origin of parents, type of occupation, marital status, job demands, job control, violence, and bullying. Statistically significant paths are shown in solid lines.

All associations for the fully adjusted model are depicted in Figure 4. There was no association found between witnessed GBH and psychological treatment (0.014, *p* = 0.408). Psychological treatment at T1 was associated with witnessed GBH at T2 (0.019, *p* = 0.012). Auto-regressive paths from T1 to T2 indicated stability for both psychological treatment (0.362, *p* = 0.000) and witnessed GBH (0.116, *p* = 0.000). The corresponding fit statistic values were RMSEA = 0.000, CFI = 1.000, SRMR = 0.000, and TLI = 1.000.

## Discussion

In the current study, we tested hypothesized and reversed longitudinal associations between experienced or witnessed GBH on the one hand, and depressive symptoms and psychological treatment on the other, between the years 2018 and 2020. We found some support for weak reversed longitudinal associations between indicators of poorer mental health (i.e., depressive symptoms and seeking psychological treatment) and future exposure to experienced and witnessed GBH but no support for the hypothesized longitudinal associations.

### Experienced GBH and poor mental health

#### Symptoms of depression

Our study sheds new light on previous findings, showing that having experienced or witnessed GBH (which may include behaviors with or without sexual content) over time led to poorer mental health (Schneider et al., 1997; Glomb et al., 1999; Munson et al., 2000; Fitzgerald and Cortina, 2017). Our results found weak effects of a reverse association, which can have several possible explanations. One may be that the results of Schneider et al. (1997), Glomb et al. (1999), Munson et al. (2000), and Fitzgerald and Cortina (2017) mostly examined health consequences of sexual harassment, whereas our outcome variable GBH include behaviors with and without sexual content. Another explanation may be that the analyses in this study were adjusted for the cross-sectional association at T2 and took stability in exposure and outcome variables across time into account, while previous studies did not. Although this is a robust method, it may also include over adjustments that could make the studied associations unnecessarily conservative. However, in a recent study, Rugulies et al. (2020) found that while depressive symptoms worsened after exposure to sexual harassment, those who were harassed were also more likely to show depressive symptoms at baseline. Unfortunately, Rugulies et al. were unable to investigate bi-directional associations between depressive symptoms and sexual harassment due to limitations in their study design. Another difference in our study is the exposure measure of GBH; the associations with mental health outcomes may differ compared to the more common exposure measure of sexual harassment. However, GBH was found prospectively associated with all-cause sickness absence and psychotropic medication in two previous studies (Blindow et al., 2021, 2022) of Swedish working populations.

#### Stressor-to-strain vs. strain-to-stressor

Our findings of weak reversed rather than hypothesized associations between GBH and mental health outcomes may be illuminated by the strain-to-stressor framework. Several studies showing reciprocal relations between job stressors and workers' well-being were summarized in a systematic review by Tang (2014). These results correspond with the strain-to-stressor-hypothesis (Zapf et al., 1996; de Lange et al., 2005) and go beyond the classic “stressor-to-strain”-effect championed by, for example, the Job Demand-Control-Support model (Karasek and Theorell, 1992). The strain-to-stressor theory argues that there is a reversed model where strain can predict an increase in experienced stressors. The results from our study suggest that experiences of poor mental health may, over time, be a risk factor for becoming a target of GBH, and the strain-to-stressor framework offers at least two models that may explain this: the “health selection” and the “perception” hypotheses. The health selection hypothesis asserts that workers in good health tend to thrive and further strengthen their occupational position, while workers with health problems risk deterioration of their employment situation, which in turn increases their negative health. The perception hypothesis posits that workers in good health are more likely to perceive their situation as positive and empowering, regardless of the actual situation, and thus feel more motivated and energized to perform well and advance into better working conditions. In contrast, workers with problems such as depressive symptoms, burnout, or emotional exhaustion will evaluate their working situation more negatively, feel less attached to their job, and perform worse as the strain on their resources further increases (de Lange et al., 2005; Tang, 2014). Following the health selection hypothesis, individuals with poorer mental health may be more likely to stay in or get into job situations and positions where gender harassment occurs to a greater extent. Another possibility, in line with the perception hypothesis, is that respondents with poorer mental health to a greater extent appraise the situation as harassing than respondents with better mental health (de Lange et al., 2005; Nielsen and Einarsen, 2012; Tang, 2014). Due to a vulnerable state, it is possible that participants with poorer mental health may have less successful coping strategies and have more difficulty activating support or calming their emotional response. Furthermore, some individuals with poor mental health may also stand out more or be socially non-conforming in a workgroup, making them perceived as easier targets of harassment. Research has shown that people suffering from depression are perceived as less agreeable and are more likely to exhibit quarrelsome behavior (Rappaport et al., 2017), which could mean that they also receive less support from bystanders when they are victimized.

#### Psychological treatment

There has historically been a disconnect between how laypersons and people experiencing harassment understand and label GBH compared to how the scientific community defines the construct (Timmerman and Bajema, 1999; Nielsen et al., 2010; Holland and Cortina, 2013; Zelin et al., 2022). There is also evidence of a reluctance to self-identify as a target of GBH for a variety of reasons (Magley and Shupe, 2005; Collinson and Collinson, 2016). All this may affect the results for those who experienced GBH and those seeking psychological treatment. The lack of a prospective association between GBH and treatment is in line with Shannon et al. (2007), but our additional results of a weak, reversed association is a novel finding. It is possible that the process of seeking psychological treatment—even if attended due to unrelated issues—helped the participants to understand and identify the offensive behavior at a later stage and explains the reversed association in our results (Crull, 1982; Woody and Perry, 1993).

### Witnessed GBH and poor mental health

When adjusted for the cross-sectional associations at both measurement points as well as for the stability in exposure and outcome variables over time, neither the hypothesized nor reversed association between witnessed GBH and depressive symptoms over time were found in our analyses. The study design may, like for experienced GBH, be the reason that the results differ from those reported in other studies (Glomb et al., 1997; Hitlan et al., 2006; Folke et al., 2020). Furthermore, in our analyses of witnessed GBH, we excluded participants who had experienced GBH; thus, the exposure measurement was more strictly defined than in previous studies. The explanation for why received psychological treatment was associated with witnessed GBH 2 years later may differ somewhat from those regarding having experienced GBH, as presented above. Witnessing GBH, as opposed to personally experiencing it, may not elicit the direct negative emotions needed to prompt psychological treatment. However, partaking in treatment, not least in formats like group therapy, may increase participants' attentiveness to and understanding of overt and covert communication between individuals outside therapy (Salisbury et al., 1986). This aligns with the findings of Wiener et al. (2005), where participants were more likely to positively identify observed harassment and discrimination when they recalled previous personal examples, priming them to spot harassment more readily.

## Strengths and limitations

### Strengths

This study is among the first to examine the longitudinal relationship between GBH and mental health outcomes with a cross-lagged panel design including both prospective and reversed associations. The analyses took several potential confounders into account and adjusted for the cross-sectional associations between the exposures and outcomes at both measurement points, thus employing a methodologically sound statistical model. To our knowledge, this is also one of the first studies to analyze people who witnessed GBH separately from those who have experienced it. Other novel contributions are the associations between GBH and having sought psychological treatment. The study was also based on a large, population-based sample.

### Limitations

This study has certain limitations. First, since the SLOSH sample includes a higher proportion of women, older workers, and employees who were born in Sweden, are married, and have a university education, the generalizability of our results may be limited due to selective attrition. The exposure variables were constructed particularly for the SLOSH study. Although this comes with limitations, the use of self-constructed single- or multi-item questions relating to harassment is not uncommon (Willness et al., 2007), and it has provided no differences in findings compared to other instruments (Sojo et al., 2016). Furthermore, it was not possible to investigate gender differences, exposure frequency, or the source of the harassment (manager, co-worker, and client/third party) due to a lack of statistical power. Furthermore, there was no information in the SLOSH questionnaire on the severity of the harassment. Another important issue to raise is that we used data from spring 2020, at the start of the COVID-19 pandemic, and the changes in working life that occurred at this time may have influenced our results, indicated by, for example, the number of exposures to GBH at work decreased between 2018 and 2020. It is possible that rates of GBH fell due to employees spending less physical time in the workplace, thus reducing the situations for harassment to take place. We could, however, see that rates of bullying and violence at the workplace increased from 2018 to 2020, suggesting that negative interpersonal incidents were a continuous issue. Additionally, individuals' perception of the harassment may have been affected by factors we did not measure in the present study. For example, the model of harm proposed by Fitzgerald et al. (1997) suggests that how people experiencing sexual- or gender-based harassment interpret and react is based not only on the frequency and severity of the harassment but also on factors such as vulnerability, attitudes, and prior experience/trauma. While we have controlled for several covariates, such as socioeconomic factors, demands, control, violence, and bullying at work, we did not have information on experiences of harassment before 2018. It also bears to remember that the items regarding depression were related to the past week. This means that there was a long time lag between the ratings of GBH and the ratings of experiences of depression. The harassment may not have persisted for that long a time, resulting in the lack of association. However, conflicting suggestions exist as researchers have proposed a time frame of at least 24 months as preferable for studying long-term effects in the case of bullying (Blomberg and Rosander, 2021), with Einarsen and Nielsen (2015) finding that reverse causation between distress and bullying tapers off for women, but not men, over a 5-year time frame. Similarly, it was found in a recent study (Blindow et al., 2022) that sexual and gender harassment was associated with an increase in psychotropic medication use during an average follow-up time of over 5 years. Finally, although our statistical model was sophisticated, in that it adjusted for cross-sectional associations between GBH and mental health outcomes in 2020, this could also be considered an over-adjustment. The associations between poor mental health and future exposure to GBH were all rather small, which could possibly be explained by said over-adjustment. Overall, using a population-based sample and sound statistical methodology, this study offers new knowledge about the complex association between workplace gender-based harassment and mental health.

## Conclusion

In a model that simultaneously measured hypothesized and reversed longitudinal associations between experienced or witnessed GBH and indications of poor mental health, we found support only for the reversed associations between poor mental health and future exposure to GBH. These results suggest a high level of complexity in the associations between gender harassment and mental health in the workplace and call for more research on bidirectional paths.

## Data availability statement

The raw data supporting the conclusions of this article will be made available by the authors, without undue reservation.

## Ethics statement

The studies involving humans were approved by the Regional Research Ethics Board in Stockholm (No: 2019-05590). The studies were conducted in accordance with the local legislation and institutional requirements. The participants provided their written informed consent to participate in this study.

## Author contributions

JP: Conceptualization, Writing—original draft, Writing—review & editing. AN: Conceptualization, Funding acquisition, Methodology, Project administration, Writing—original draft, Writing—review & editing. PP: Data curation, Formal analysis, Methodology, Writing—review & editing.
