# Supplementary material for: Bi-directional associations between gender-based harassment at work, psychological treatment and depressive symptoms
Source: Front Psychol. 2023 Nov 29;14:1278570. doi: 10.3389/fpsyg.2023.1278570 (PMC10716254; doi:10.3389/fpsyg.2023.1278570)
Supplement: Supplementary file 1 [file Data_Sheet_1.PDF]

Appendix

Path coefficients between gender-based harassment and outcome variables (Data: SLOSH 2018-2020, n=6298)

	Model 1 β (95% CI)	p	Model 2 β (95% CI)	p	Model 3 β (95% CI)	p	Model 4 β (95% CI)	p
Experienced GBH - Depressive symptoms								
Depress T1 - Depress T2	0.595 (0.576 to 0.614)	0.000	0.583 (0.563 to 0.602)	0.000	0.573 (0.553 to 0.593)	0.000	0.573 (0.552 to 0.593)	0.000
GBH-E T1 - Depress T2	0.457 (-0.016 to 0.931)	0.058	0.142 (-0.342 to 0.627)	0.565	0.078 (-0.412 to 0.569)	0.755	0.029 (-0.477 to 0.536)	0.909
Depress T1 - GBH-E T2	0.002 (0.001 to 0.003)	0.000	0.002 (0.001 to 0.003)	0.000	0.002 (0.001 to 0.003)	0.000	0.002 (0.001 to 0.003)	0.000
GBH-E T1 - GBH-E T2	0.243 (0.224 to 0.261)	0.000	0.235 (0.215 to 0.254)	0.000	0.235 (0.215 to 0.255)	0.000	0.221 (0.201 to 0.241)	0.000
Experienced GBH - Psychological treatment								
Treat T1 - Treat T2	0.389 (0.367 to 0.411)	0.000	0.365 (0.342 to 0.388)	0.000	0.359 (0.336 to 0.382)	0.000	0.360 (0.337 to 0.383)	0.000
GBH-E T1 - Treat T2	0.054 (0.016 to 0.092)	0.005	0.034 (-0.005 to 0.072)	0.088	0.026 (-0.13 to 0.065)	0.193	0.031 (-0.001 to 0.071)	0.139
Treat T1 - GBH-E T2	0.017 (0.006 to 0.028)	0.002	0.015 (0.004 to 0.026)	0.010	0.014 (0.002 to 0.025)	0.018	0.013 (0.001 to 0.024)	0.027
GBH-E T1 x GBH-E T2	0.249 (0.231 to 0.268)	0.000	0.241 (0.222 to 0.261)	0.000	0.240 (0.221 to 0.260)	0.000	0.226 (0.206 to 0.246)	0.000
Witnessed GBH - Depressive symptoms								
Depress T1 - Depress T2	0.593 (0.573 to 0.613)	0.000	0.580 (0.560 to 0.601)	0.000	0.571 (0.550 to 0.593)	0.000	0.570 (0.549 to 0.592)	0.000
GBH-W T1 - Depress T2	0.123 (-0.291 to 0.536)	0.561	0.064 (-0.360 to 0.488)	0.766	0.012 (-0.416 to 0.438)	0.957	0.026 (-0.403 to 0.455)	0.906
Depress T1 - GBH-W T2	0.001 (0.000 to 0.002)	0.011	0.001 (0.000 to 0.002)	0.006	0.001 (-0.000 to 0.002)	0.068	0.001 (-0.000 to 0.002)	0.082
GBH-W T1 - GBH-W T2	0.123 (0.102 to 0.144)	0.000	0.118 (0.097 to 0.139)	0.000	0.115 (0.094 to 0.137)	0.000	0.114 (0.092 to 0.135)	0.000
Witnessed GBH - Psychological treatment								
Treat T1 - Treat T2	0.389 (0.366 to 0.412)	0.000	0.367 (0.344 to 0.391)	0.000	0.361 (0.338 to 0.384)	0.000	0.362 (0.338 to 0.386)	0.000
GBH-W T1 - Treat T2	0.020 (-0.013 to 0.053)	0.243	0.014 (-0.19 to 0.04)	0.397	0.011 (-0.022 to 0.045)	0.509	0.014 (-0.19 to 0.048)	0.408
Treat T1 - GBH-W T2	0.023 (0.009 to 0.038)	0.002	0.023 (0.080 to 0.038)	0.003	0.021 (0.006 to 0.036)	0.006	0.019 (0.004 to 0.034)	0.012
GBH-W T1 - GBH-W T2	0.125 (0.104 to 0.146)	0.000	0.121 (0.100 to 0.142)	0.000	0.117 (0.096 to 0.138)	0.000	0.116 (0.094 to 0.137)	0.000

GBH-E =Experienced gender-based harassments; GBH-W = Witnessed gender-based harassments; Depress = Depressive symptoms; Treat = Psychological treatment; T1 = 2018; T2 = 2020.

Model 1: Crude model.

Model 2: Adjusted for age, gender, education, income, country of birth, type of occupation, marital status, origin of parents.

Model 3: Model 2 + job demands, job control.

Model 4: Model 3 + violence, bullying.
